# Effects of sleep deprivation on heart rate variability: a systematic review and meta-analysis

**DOI:** 10.3389/fneur.2025.1556784

**Published:** 2025-08-14

**Authors:** Suling Zhang, Xiaodan Niu, Jinke Ma, Xin Wei, Jun Zhang, Weiping Du

**Affiliations:** ^1^Department of Public Basic Teaching, Xi’an Academy of Fine Arts, Institute of Body-Medicine Integration, Xi’an, China; ^2^Xi’an Jiaotong University Sports Centre, Xi’an, China; ^3^School of Software, Xi’an Jiaotong University, Xi’an, China; ^4^College of Humanities Education, Xi’an Eurasian College, Xi’an, China; ^5^School of Physical Education and Sport, Ningxia Normal University, Guyuan, China

**Keywords:** sleep deprivation, HRV, autonomic nervous, systematic review, meta-analysis

## Abstract

**Background:**

Sleep deprivation is prevalent in high-pressure environments and among shift workers, and may contribute to autonomic nervous system (ANS) dysregulation, contributing to cardiovascular diseases, mood disorders, and cognitive impairment. Heart rate variability (HRV), an important indicator of ANS function, reflects fluctuations in sympathetic and parasympathetic activity and is commonly used to assess the autonomic effects of sleep deprivation. However, existing studies exhibit considerable heterogeneity due to inconsistencies in HRV measurement methods, variations in deprivation duration, and inadequate control of confounding factors.

**Objective:**

This study aimed to comprehensively evaluate the impact of sleep deprivation on HRV through a systematic review and meta-analysis of randomized controlled trials (RCTs), to elucidate the potential mechanisms underlying sleep deprivation-induced cardiac autonomic dysfunction, and to provide insights for optimizing sleep-related interventions and preventing cardiovascular disease.

**Methods:**

A systematic search was conducted in PubMed, Embase, CNKI, Wanfang, and VIP databases for RCTs investigating the effects of sleep deprivation on HRV, covering the period from January 2010 to May 2024. The Cochrane Risk of Bias tool was used for methodological quality assessment. Meta-analyses were performed using Review Manager 5.4 and Stata 17.0 software.

**Results:**

A total of 11 eligible studies involving 549 participants were included. The meta-analysis revealed that: (1) In the time domain, sleep deprivation was associated with a non-significant reduction in SDNN (*p* > 0.05), while RMSSD showed a significant decrease (*p* < 0.05). (2) In the frequency domain, both LF and LF/HF significantly increased after sleep deprivation (*p* < 0.05), whereas HF showed a decreasing trend that did not reach statistical significance (*p* > 0.05).

**Conclusion:**

This meta-analysis indicates that sleep deprivation may impair cardiac autonomic function, as evidenced by decreased RMSSD and increased LF and LF/HF, suggesting sympathetic predominance and vagal suppression. However, changes in other HRV indices such as SDNN and HF were not statistically significant. These findings imply a potential disruption of the dynamic balance between sympathetic and parasympathetic activity following sleep deprivation. Future research should adopt standardized HRV measurement protocols to validate these findings and further explore the underlying physiological mechanisms. This study provides important evidence for understanding the dynamic changes in autonomic function associated with sleep deprivation.

**Systematic review registration:**

https://inplasy.com/projects/, identifier INPLASY202560023.

## 1 Introduction

Sleep deprivation has been extensively studied and is known to be closely associated with the development of various chronic metabolic diseases, including hypertension, diabetes, cardiovascular disease, and obesity (1, 2). Sleep deprivation leads to multiple physiological disturbances that predispose individuals to metabolic disorders. These include dysregulation of appetite-related hormones, such as increased ghrelin and decreased leptin levels, which promote weight gain (3, 4), and activation of the hypothalamic-pituitary-adrenal (HPA) axis, resulting in elevated cortisol secretion (1, 5). These neuroendocrine disruptions impair glucose metabolism, reduce insulin sensitivity, and compromise endothelial function. Consequently, epidemiological evidence shows that individuals who sleep less than 6 h per day exhibit a significantly increased risk of developing hypertension and other chronic metabolic conditions (6, 7). Chronic sleep deprivation can also impair the function of metabolically relevant organs such as the liver and pancreas, leading to reduced insulin secretion and endothelial dysfunction, thereby increasing the risk of diabetes and cardiovascular disease (2, 5, 8), and further burdening individual health (9).

In the domain of mental health, numerous studies have confirmed a strong association between sleep deficiency and emotional disturbances. Insufficient sleep has been linked to heightened symptoms of depression and anxiety, along with impairments in mood regulation and cognitive performance (10, 81). Li (11) reported that sleep loss may exacerbate negative emotions, thereby worsening emotional well-being. Chang et al. (12) further highlighted a bidirectional relationship between sleep deprivation and affective disorders, suggesting that interventions should simultaneously address both emotional health and sleep quality (82).

Sleep deprivation has also been shown to weaken immune responses. Specifically, it reduces T-cell activity and inhibits the secretion of key cytokines such as IL-1β and TNF-α, which are critical for host defense (13). Consistently, Andani and Arif (14) reported that chronic sleep loss may contribute to decreased mood, reduced energy, and weakened immune defense, increasing susceptibility to infections and disease (84).

Sleep deprivation also significantly impacts cardiac autonomic nervous system (ANS) function. Research has shown that sleep deprivation may alter the density of β1-adrenergic receptors in the heart and reduce the release of vagal neurotransmitter acetylcholine, thereby increasing the risk of arrhythmia (13, 80). Xue et al. (15) confirmed that 24-h sleep deprivation markedly elevates sympathetic nervous system activity, leading to impaired cardiovascular function and increased cardiovascular risk. Similarly, Chen et al. (86) demonstrated that rapid eye movement (REM) sleep deprivation in rats led to significant deterioration in cardiac autonomic regulation, suggesting that sleep quality directly affects cardiac health. Further evidence from microneurography studies in healthy individuals revealed increased sympathetic nerve activity after sleep deprivation, which is significantly associated with arrhythmic risk (16). These findings underscore the clinical relevance of managing sleep patterns to protect cardiovascular health.

Heart rate variability (HRV) is an important quantitative indicator of cardio-autonomic nervous system (ANS) activity and is widely used in sleep research, particularly for analyzing sleep quality and associated health risks (17, 18, 78). Time-domain metrics of HRV, such as the standard deviation of normal-to-normal intervals (SDNN), have been found to correlate significantly with cardiovascular events. A reduction in SDNN is closely associated with increased cardiovascular mortality, suggesting that HRV can serve as an early warning signal for autonomic dysfunction (19, 85).

Frequency-domain indicators of HRV reflect the regulatory states of the sympathetic and parasympathetic nervous systems. Low-frequency power (LF) reflects a mix of both sympathetic and parasympathetic modulation, while high-frequency power (HF) primarily represents parasympathetic (vagal) activity (20). The LF/HF ratio has often been interpreted as a marker of sympathovagal balance. However, this interpretation has been questioned, as the physiological basis of the LF/HF ratio is influenced by numerous confounding factors, including respiratory rate, heart rate, and baroreflex sensitivity. Consequently, over-reliance on LF/HF as a direct measure of autonomic balance should be avoided (21).

Although the interpretation of the LF/HF ratio as an index of sympathovagal balance remains controversial, numerous studies have consistently shown that sleep deprivation significantly alters heart rate variability (HRV) patterns, reflecting autonomic dysregulation (22). These alterations are evident in both frequency-domain indices (e.g., LF, HF, LF/HF) and time-domain measures. In contrast, high-quality sleep is typically associated with elevated HF values and enhanced parasympathetic tone, indicating that adequate sleep contributes to the maintenance of autonomic stability (20). Collectively, these findings underscore the significant influence of sleep status on autonomic nervous system activity and highlight the clinical utility of HRV in evaluating the physiological consequences of insufficient sleep (76).

Despite its utility, existing HRV research on sleep deprivation is characterized by significant methodological heterogeneity, including inconsistencies in measurement techniques, variation in deprivation protocols, and limited control of confounding variables. To address these limitations, the present study conducted a systematic review and meta-analysis of randomized controlled trials (RCTs) assessing the effects of sleep deprivation on five standard HRV indices (SDNN, RMSSD, LF, HF, LF/HF). This approach aimed to synthesize current evidence, identify consistent patterns, and provide a more reliable understanding of the autonomic consequences of sleep loss.

## 2 Methods

### 2.1 Protocol registration

This systematic review was registered with the International Platform of Registered Systematic Review and Meta-analysis Protocols (INPLASY) under the registration number INPLASY202560023 (DOI: 10.37766/inplasy2025.6.0023).

### 2.2 Literature search strategy

A comprehensive search was conducted in the following databases: PubMed, Embase, CNKI (China National Knowledge Infrastructure), Wanfang, and VIPro. The focus was on identifying randomized controlled trials (RCTs) investigating the effects of sleep deprivation on heart rate variability (HRV). The selection of RCTs was based on the following rationale: First, RCTs utilize random assignment to effectively control for confounding variables, thereby providing higher internal validity when assessing the causal relationship between sleep deprivation and changes in HRV. Second, HRV is highly susceptible to a variety of influences, including environmental conditions, body posture, time of day, food intake, and psychological state. RCT designs allow for the standardization of these factors, thereby improving the comparability across studies.

The search covered studies published between January 2010 and May 2024. The actual database searches were executed in May 2024. Both subject headings and free-text terms were used in combination to enhance search sensitivity. The detailed search strategy is presented in Table 1.

**Table 1:** Example of database search strategy.

Database	Search strategy	Search strategy (keywords/subject terms)
PubMeD and Embase	Combination of MeSH terms and free-text terms	((((sleep deprivation) OR (Deprivation, Sleep)) OR (REM Sleep Deprivation)) OR (Deprivation, REM Sleep)) OR (Sleep Deprivation, REM)) OR (Sleep Insufficiency)) OR (Insufficiencies, Sleep)) OR (Insufficiency, Sleep)) OR (Sleep Insufficiencies)) OR (Insufficient Sleep)) OR (Sleep, Insufficient)) OR (Inadequate Sleep)) OR (Sleep, Inadequate)) OR (Sleep Fragmentation)) OR (Fragmentation, Sleep)) OR (Insufficient Sleep Syndrome)) OR (Insufficient Sleep Syndromes)) OR (Syndrome, Insufficient Sleep)) OR (Sleep Debt)) AND (heart rate variability)
CNKI	Combination of MeSH terms and free-text terms	((Topic: sleep deprivation) OR (Topic: sleep loss)) AND ((Topic: autonomic nervous system) OR (Topic: heart rate variability) OR (Topic: HRV))
Wanfang and Wipro	Combination of MeSH terms and free-text terms	((Topic: sleep deprivation) OR (Topic: sleep loss)) AND ((Topic: autonomic nervous system) OR (Topic: heart rate variability) OR (Topic: HRV))

### 2.3 Inclusion and exclusion criteria

#### 2.3.1 Inclusion criteria

(1) Study type: Only randomized controlled trials (RCTs) were included. (2) Participants: Healthy individuals without any diagnosed diseases. (3) Intervention: The experimental group received sleep deprivation as the intervention; the control group maintained normal sleep without any intervention.

#### 2.3.2 Exclusion criteria

(1) Conference abstracts and studies for which the full text was unavailable. (2) Studies were excluded if they did not report HRV-related outcome indicators clearly (e.g., missing means and standard deviations, absence of control group data, or data presented only in figures without numerical values), which made it impossible to extract valid endpoint data. (3) Duplicate publications and review articles. (4) Studies involving animal experiments, patient populations, or pharmacological interventions.

### 2.4 Outcome measures

Heart rate variability (HRV) includes time-domain, frequency-domain, and nonlinear indices. In this study, five widely accepted “gold-standard” HRV metrics were selected for analysis, based on previous literature (23).

Time-domain analysis primarily uses statistical methods to assess the variability in successive R–R intervals. The most commonly used indicators include the standard deviation of normal-to-normal intervals (SDNN) and the root mean square of successive differences between adjacent R–R intervals (RMSSD) (24). Although RMSSD is highly correlated with SD1 derived from Poincaré plot analysis—a nonlinear method—and can sometimes be considered approximately equivalent, other geometric HRV metrics such as the HRV triangular index, the triangular interpolation of NN interval histogram (TINN), and SD2 should be classified and interpreted independently of time-domain measures (25).

Frequency-domain analysis involves decomposing electrocardiographic signals into sinusoidal waves of different amplitudes and frequencies, either via analog or digital signal processing techniques. Common frequency-domain indices include low-frequency power (LF), high-frequency power (HF), and the LF/HF ratio (26). While the LF/HF ratio has been widely used as an indicator of sympathovagal balance, its physiological interpretation has been increasingly questioned due to potential oversimplification and methodological limitations. As Billman (21) emphasized, the validity of LF/HF relies on several assumptions: that LF reflects mainly sympathetic activity and HF reflects parasympathetic activity. However, evidence suggests that LF is modulated by both sympathetic and parasympathetic influences, and its correlation with actual sympathetic nerve activity is inconsistent. Moreover, non-neural factors such as respiratory frequency, heart rate levels, and baroreflex sensitivity can significantly affect both LF and HF values (21). Therefore, the interpretation of the LF/HF ratio should be approached with caution and should not be used as a simplistic marker of cardio-autonomic balance (83).

### 2.5 Quality assessment of included studies

At present, several tools are available for evaluating the methodological quality of studies, including the Cochrane Risk of Bias tool, the modified Jadad scale, the Newcastle–Ottawa Scale (NOS), the MINORS criteria, and the QUADAS instrument.

Given that all studies included in this review were randomized controlled trials (RCTs), the Cochrane Risk of Bias (RoB) tool—developed to assess methodological quality across multiple domains—was selected for quality assessment (27).

The Cochrane RoB tool evaluates methodological quality across six domains: (1) random sequence generation, (2) allocation concealment, (3) blinding, (4) completeness of outcome data, (5) selective reporting, and (6) other potential sources of bias.

Based on the number of domains fully met, studies were classified into three levels of methodological quality (28): Grade A (low risk of bias): Studies that fully satisfied four or more domains; Grade B (moderate risk of bias): Studies that fully satisfied two or three domains; Grade C (high risk of bias): Studies that satisfied only one or none of the domains.

### 2.6 Study selection and data extraction

The initial screening of the retrieved studies was conducted using EndNote X9 software (Clarivate Analytics, Philadelphia, PA, United States). For each included study, a standardized data extraction form was used to collect the following information: (1) basic characteristics (title, author, publication year, region); (2) subject characteristics (sample size, gender, health status, and duration of sleep deprivation); and (3) reported outcome indicators.

Data extraction was performed independently by two researchers. After completion, the extracted data were cross-checked for consistency. In cases of disagreement, the two researchers discussed the discrepancies. If a consensus could not be reached, a third reviewer was consulted to determine whether the study should be included.

### 2.7 Statistical analysis

Meta-analyses were conducted using Review Manager (RevMan) version 5.4 and Stata version 17.0 to calculate the pooled effect sizes from the included studies. As the outcomes were continuous variables, effect sizes were expressed as standardized mean differences (SMDs) with corresponding 95% confidence intervals (CIs).

Assessment of heterogeneity was performed using both the chi-squared (*χ*^2^) test and the *I*^2^ statistic: If *p* < 0.05 and *I*^2^ ≥ 50%, significant heterogeneity was indicated, and a random-effects model was applied. If *p* > 0.05 and *I*^2^ < 50%, heterogeneity was considered low, and a fixed-effects model was used.

In cases of high heterogeneity, subgroup analyses or sensitivity analyses were conducted to explore potential sources of variability. Egger’s regression test was performed to detect small-study effects, which may indicate potential publication bias.

## 3 Results

### 3.1 Literature search results

**Figure 1:**
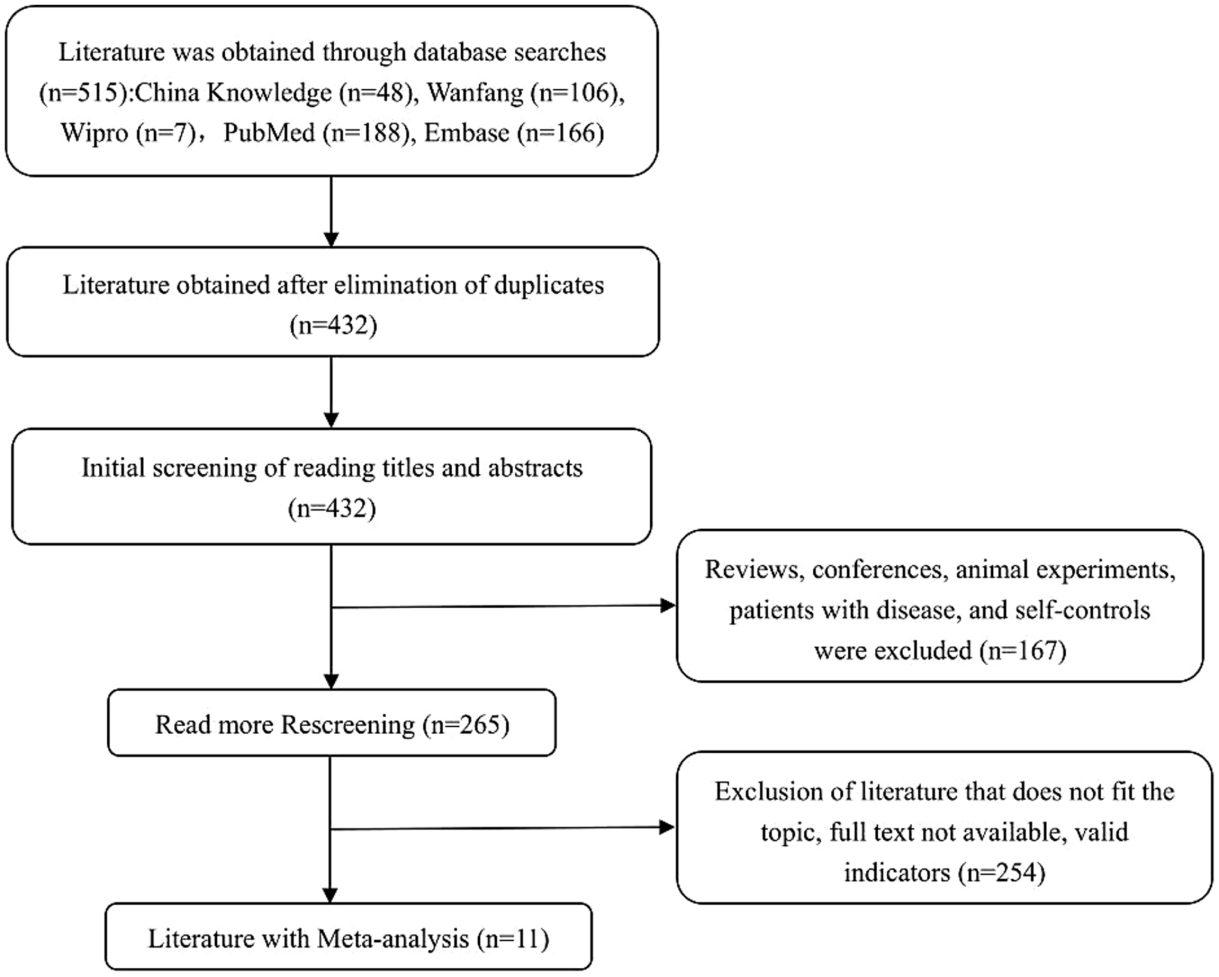
Literature screening flowchart.

A preliminary search across both Chinese and English databases yielded a total of 515 articles, including 161 in Chinese and 354 in English. All references were imported into EndNote software for screening. After removing 83 duplicates, 167 records were excluded for being reviews, conference papers, animal experiments, studies involving patients with existing diseases, or self-controlled trials. An additional 254 articles were excluded after abstract screening due to irrelevance to the study topic, unavailability of full text, or lack of extractable outcome indicators. Ultimately, 11 eligible studies were included in the final meta-analysis. The detailed study selection process is presented in Figure 1, following the PRISMA 2020 guidelines (29).

### 3.2 Basic characteristics of included studies

A total of 11 studies were included, consisting of four articles published in Chinese and seven in English. The study populations were from various backgrounds, including healthy individuals, shift workers, astronauts, and university students. The basic characteristics of the included studies are summarized in Table 2.

**Table 2:** Basic characteristics of included studies.

Author and year	District	Age	Genders	Design	Research object	Sample size		Deprivation of time	Deprivation of location	Indicators of outcome
						SD group	Control group			
Vierra et al. (1)	Thailand	19–25	Male/female	RCT	Healthy population	22	21	<7 h	Laboratory	a, b, c, d, e
Liu et al. (69)	China	18–30	Male	RCT	Astronauts	6	6	72 h	Laboratory	c, d
Tobaldini et al. (41)	Italian	18–40	Male/female	RCT	Healthy population	8	9	6 h	Laboratory	d, e
Wehrens et al. (42)	UK	25–45	Male	RCT	Shift worker	11	14	8 h	Laboratory	b, c, d, e
Morales et al. (70)	Spanish	26–55	Male/female	RCT	Resident doctor	40	18	24 h	Hospital	c, d, e
van Leeuwen et al. (71)	Suomi	19–29	Male	RCT	Healthy population	15	8	<4 h	Laboratory	c, d, e
Kunikullaya et al. (72)	Indian	18–55	Male/female	RCT	Shift worker	36	36	8 h	Corporation	c, d, e
Li et al. (13)	China	35–36	Male	RCT	Miners	36	36	24 h	Laboratory	e
Ma et al. (73)	China	19–52	Male/female	RCT	Naval personnel	78	97	24 h	Laboratory	a, b
Guan et al. (74)	China	21–24	Male	RCT	University student	20	20	24 h	Laboratory	a, b, c, d, e
Wu et al. (75)	China	24–29	Male	RCT	University student	6	6	12 h	Laboratory	a, c, d, e

RCT indicates randomized controlled trial. (a) Denotes SDNN (standard deviation of R–R intervals). (b) Denotes RMSSD (root mean square of successive differences between adjacent R–R intervals). (c) Represents LF (low-frequency power). (d) Represents HF (high-frequency power). (e) Represents LF/HF (the ratio of low-frequency power to high-frequency power).

### 3.3 Quality assessment of included studies

**Figure 2:**
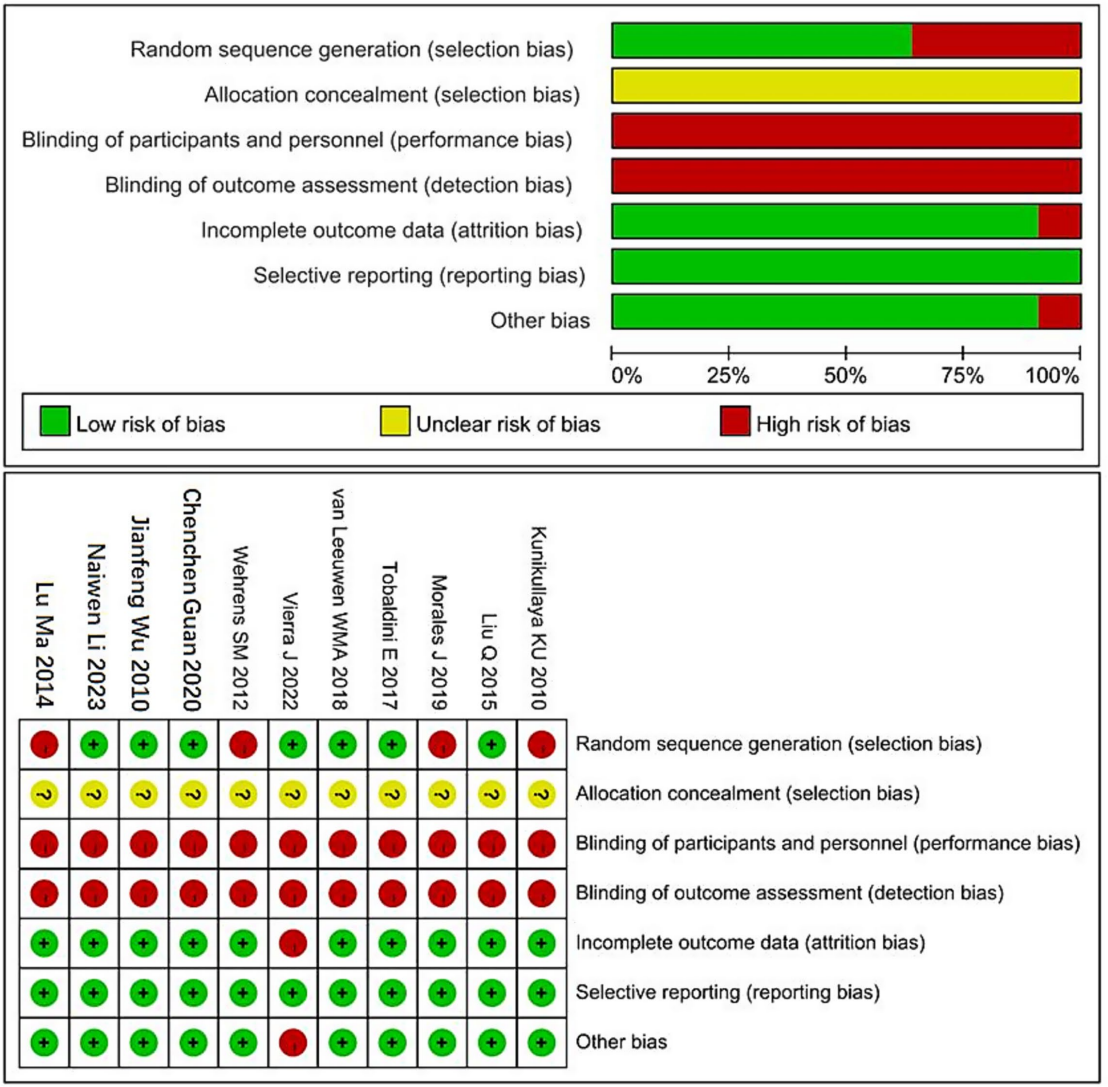
Cochrane risk of bias assessment chart. “+” Indicates low risk of bias. “−” Indicates high risk of bias. “?” Indicates unclear risk of bias.

The methodological quality of the included studies was assessed using the Cochrane Risk of Bias tool. Among the 11 included studies, 7 reported the use of random sequence generation, 10 had complete outcome data, and none exhibited selective reporting. One study was identified as having other potential sources of bias. Detailed results of the quality assessment are shown in Figure 2.

### 3.4 Meta-analysis results

#### 3.4.1 SDNN

**Figure 3:**
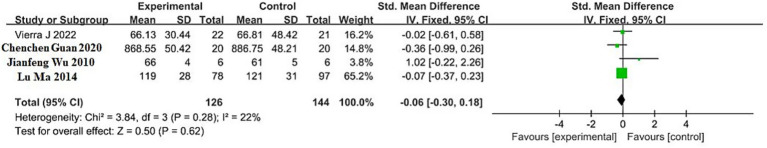
Forest plot of SDNN changes between sleep deprivation and control groups.

The pooled result for SDNN (standard deviation of normal-to-normal intervals, an indicator of overall HRV) was based on 4 studies, with 126 participants in the sleep deprivation groups and 144 in the control groups. As shown in Figure 3, the heterogeneity analysis yielded *χ*^2^ = 3.84, df = 3, *p* = 0.28 (>0.05), and *I*^2^ = 22% (<50%), indicating acceptable homogeneity among studies. Therefore, a fixed-effects model was applied.

The meta-analysis yielded *Z* = 0.50, *p* = 0.62, with an SMD of −0.06 and a 95% confidence interval (−0.30, 0.18), indicating that sleep deprivation had no statistically significant effect on SDNN. The detailed results are illustrated in the forest plot in Figure 3.

#### 3.4.2 RMSSD

**Figure 4:**
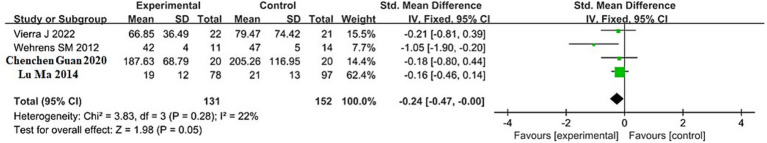
Forest plot of RMSSD changes between sleep deprivation and control groups.

The pooled result for RMSSD (root mean square of successive differences, an index of parasympathetic activity) was based on 4 studies, including 131 participants in the sleep deprivation groups and 152 in the control groups. As shown in Figure 4, the heterogeneity analysis yielded *χ*^2^ = 3.83, df = 3, *p* = 0.28, and *I*^2^ = 22% (<50%), indicating low heterogeneity; therefore, a fixed-effects model was applied.

The meta-analysis showed *Z* = 1.98, *p* < 0.05, with an SMD of −0.24 and a 95% confidence interval (−0.47, −0.00). These results indicate that sleep deprivation significantly reduces RMSSD. The 95% confidence interval lies entirely to the left of the null line, suggesting that the RMSSD values in the sleep deprivation groups were significantly lower than those in the control groups. Detailed results are shown in the forest plot in Figure 4.

#### 3.4.3 LF

**Figure 5:**
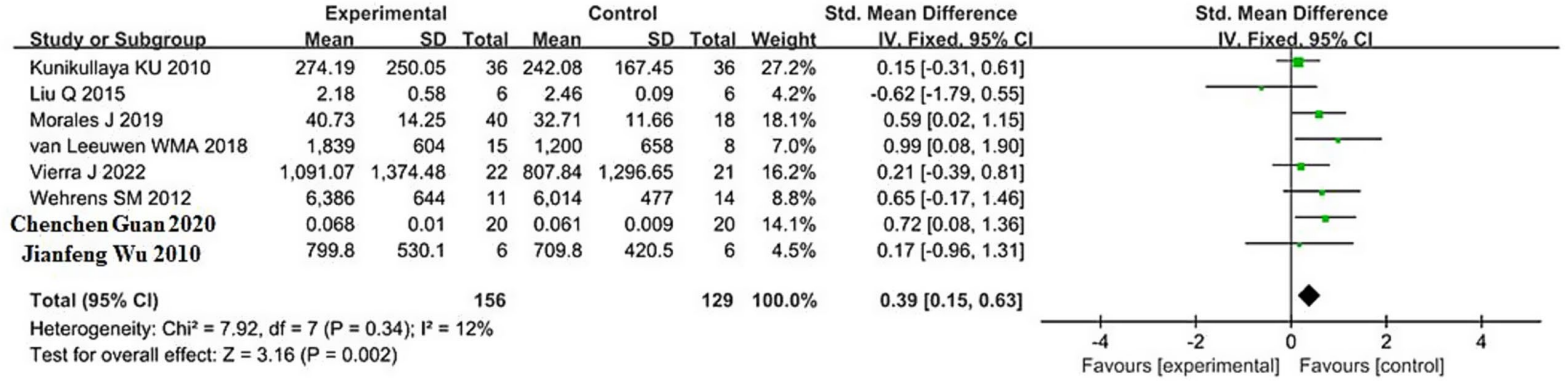
Forest plot of LF changes between sleep deprivation and control groups.

The pooled result for LF (low-frequency power, reflecting both sympathetic and parasympathetic modulation) was based on 8 studies, with 156 participants in the sleep deprivation groups and 129 in the control groups. As shown in Figure 5, the heterogeneity test yielded *χ*^2^ = 7.92, df = 7, *p* = 0.34, and *I*^2^ = 12% (<50%), indicating low heterogeneity; thus, a fixed-effects model was applied.

The meta-analysis produced *Z* = 3.16, *p* = 0.002, with an SMD of 0.39 and a 95% confidence interval of (0.15, 0.63), suggesting a significant increase in LF following sleep deprivation. The 95% confidence interval lies entirely to the right of the null line, indicating that LF values in the sleep deprivation group were significantly higher than those in the control group.

Detailed results are presented in the forest plot in Figure 5.

#### 3.4.4 HF

**Figure 6:**
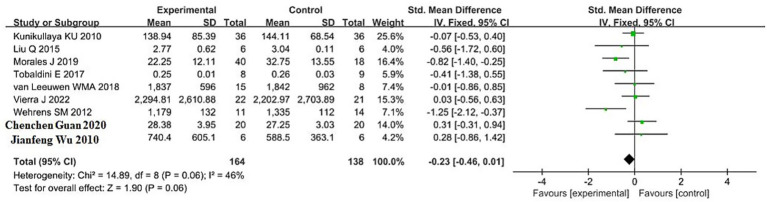
Forest plot of HF changes between sleep deprivation and control groups.

The pooled result for HF (high-frequency power, primarily reflecting parasympathetic activity) was based on 9 studies, including 164 participants in the sleep deprivation groups and 138 in the control groups. The results in Figure 6 show that *χ*^2^ = 14.89 (df = 8, *p* = 0.06) and *I*^2^ = 46% (<50%), indicating acceptable homogeneity. Therefore, a fixed-effects model was applied. The pooled standardized mean difference (SMD) was −0.23 (95% CI, −0.46 to 0.01), with a *Z* value of 1.90 and *p* = 0.06. These findings suggest that sleep deprivation was associated with a non-significant reduction in HF. Although the confidence interval lies mostly to the left of the null line, it crosses zero, indicating uncertainty in the direction and significance of the effect.

#### 3.4.5 LF/HF

**Figure 7:**
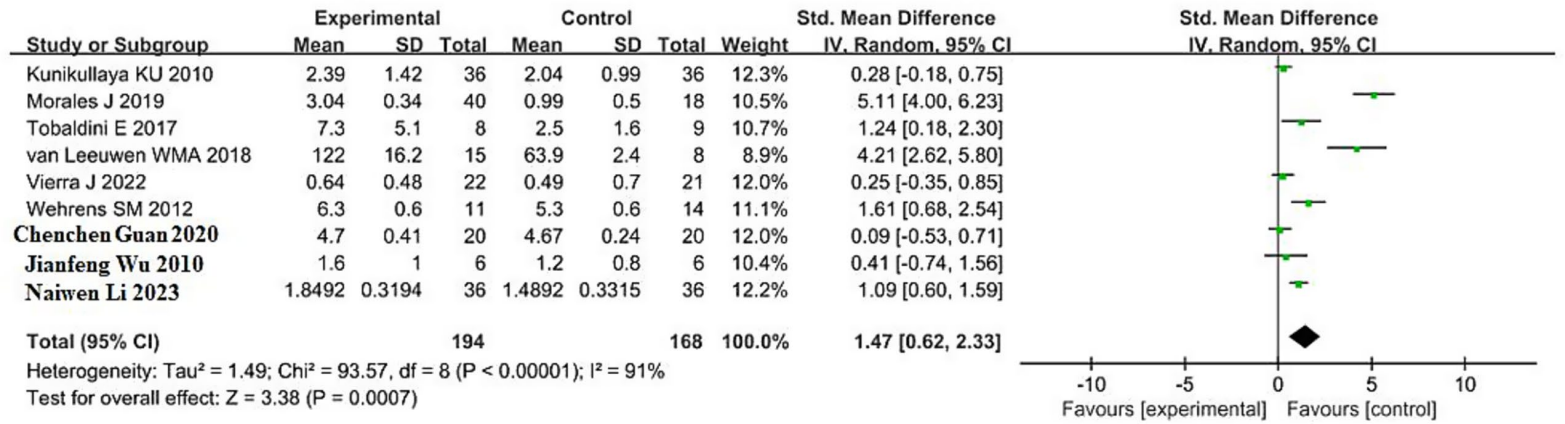
Forest plot of LF/HF changes between sleep deprivation and control groups.

A total of 9 studies reported the effects of sleep deprivation on the LF/HF ratio, including 194 participants in the sleep deprivation groups and 168 in the control groups. As shown in Figure 7, the heterogeneity test yielded *χ*^2^ = 93.57, df = 8, *p* < 0.00001, and *I*^2^ = 91%, indicating substantial heterogeneity among the included studies. Therefore, a random-effects model was used for the meta-analysis.

The analysis produced *Z* = 3. 38, *p* = 0.0007, with an SMD of 1.47 and a 95% confidence interval of (0.62, 2.33), indicating that sleep deprivation significantly increased the LF/HF ratio. The 95% confidence interval lies entirely to the right of the null line, suggesting that the LF/HF values in the sleep deprivation group were significantly higher than those in the control group. Detailed results are illustrated in the forest plot in Figure 7.

### 3.5 Subgroup analysis results

To explore potential sources of heterogeneity, subgroup analyses were conducted for the LF/HF outcome indicator using two covariates: sleep deprivation duration and type of study population. For sleep deprivation duration, studies were divided into two subgroups: <24 h and ≥24 h. For participant type, five subgroups were defined: healthy individuals, shift workers, university students, resident physicians, and miners.

As shown in Table 3, participant type appears to be a major source of heterogeneity. Among the subgroups, university students exhibited the lowest heterogeneity (*I*^2^ = 0%), whereas healthy individuals showed the highest heterogeneity (*I*^2^ = 91%). Moreover, the effect size in the healthy population subgroup was greater than that in the university student subgroup, suggesting greater variability in cardio-autonomic responses among the broader healthy population.

**Table 3:** Subgroup analysis of the effect of sleep deprivation on LF/HF.

Subgroup	Classifications	Number of publications/article	Sample size/person	Combined effect size			Heterogeneity test	
				SMD	95% CI	*p*	*I*^2^ (%)	*p*
Deprivation of time	<24 h	6	192	1.15	0.32, 1.97	0.007	83	<0.0001
	≥24 h	3	170	2.04	−0.14, 4.21	0.07	97	<0.00001
Research object	Healthy population	3	83	1.77	−0.18, 3.73	0.08	91	<0.0001
	Shift worker	2	97	0.88	−0.41, 2.18	0.18	84	0.01
	University student	2	52	0.16	−0.39, 0.71	0.57	0	0.63
	Resident doctor	1	58	5.11	4.00, 6.23	<0.00001	—	—
	Miners	1	72	1.09	0.60, 1.59	<0.0001	—	—

### 3.6 Small-study effect analysis

Egger’s regression tests were performed using the “metabias” command to assess potential small-study effects for each of the HRV outcome indicators included in this meta-analysis. The results were as follows: SDNN (*t* = 0.85, *p* = 0.484), RMSSD (*t* = −1.41, *p* = 0.295), LF (*t* = −0.07, *p* = 0.945), HF (*t* = −0.51, *p* = 0.624), LF/HF (*t* = 2.33, *p* = 0.053).

These findings suggest that no significant small-study effects were detected for any of the outcome indicators, although the LF/HF result was close to the significance threshold. Detailed results are presented in Table 4.

**Table 4:** Results of Egger’s regression analysis.

Indicators of outcome	Standard efficiency	Regression coefficient	Standard error	t	*p* > |t|	95% CI
SDNN	Slope	−0.326	0.350	−0.93	0.450	[−1.831, 1.180]
	Bias	1.213	1.424	0.85	0.484	[−4.916, 7.342]
RMSSD	Slope	0.163	0.314	0.52	0.655	[−1.188, 1.514]
	Bias	−1.835	1.304	−1.41	0.295	[−7.448, 3.777]
LF	Slope	0.431	0.499	0.86	0.421	[−0.791, 1.652]
	Bias	−0.103	1.435	−0.07	0.945	[−3.614, 3.409]
HF	Slope	0.056	0.592	0.09	0.927	[−1.345, 1.456]
	Bias	−0.848	1.655	−0.51	0.624	[−4.763, 3.066]
LF/HF	Slope	−1.103	0.921	−1.20	0.270	[−3.281, 1.074]
	Bias	5.960	2.559	2.33	0.053	[−0.090, 12.011]

## 4 Discussion

### 4.1 Methodological quality of included studies

A total of 11 studies were included in this meta-analysis. Among them, 8 studies were rated as Grade A and 5 as Grade B, indicating an overall acceptable methodological quality. Given the potential harm of sleep deprivation to human subjects, all participants signed informed consent forms and voluntarily participated in the experiments. As a result, it was difficult to implement full blinding procedures in most of the included studies.

The pooled results demonstrated statistical significance with low heterogeneity, suggesting that the findings are methodologically robust and likely to be reliable.

### 4.2 Effects of sleep deprivation on heart rate variability

This study systematically reviewed and meta-analyzed the effects of sleep deprivation on heart rate variability (HRV) indicators. Regarding time-domain HRV indices, the results showed that while SDNN exhibited a downward trend following sleep deprivation, the change was not statistically significant (SMD = −0.06, 95% CI: −0.30 to 0.18, *p* = 0.62). In contrast, RMSSD showed a significant reduction (SMD = −0.24, 95% CI: −0.47 to −0.00, *p* < 0.05). This finding is consistent with previous studies, which have highlighted that sleep deprivation may contribution to a decline in parasympathetic activity, thereby impairing autonomic nervous system (ANS) function (30, 31).

RMSSD is widely recognized as a key metric for evaluating parasympathetic regulation. A significant reduction in RMSSD indicates that parasympathetic activity may be suppressed during acute physiological stress, such as sleep deprivation (32, 33). Research has shown that sleep deprivation may activate the hypothalamic-pituitary-adrenal (HPA) axis, resulting in the release of catecholamines, which in turn inhibits vagal activity to the heart (34). This shift may also be associated with hormonal imbalances, including increased levels of ghrelin and decreased levels of leptin, which are known to regulate appetite and energy balance (35, 79).

Other studies have linked short sleep duration, poor sleep efficiency, and insomnia symptoms to increased cardio-autonomic tone, typically characterized by reduced parasympathetic tone and enhanced sympathetic tone. Epidemiological research has reported that partial sleep deprivation for as few as five consecutive nights can significantly decrease vagal activity, increase overall sympathetic output, and impair endothelial function (36). These physiological changes may, in part, explain the observed association between sleep deprivation and elevated cardiovascular risk (37–39). For instance, increased sympathetic activity under sleep-deprived conditions has been associated with elevated heart rate and vasoconstriction, which may alter vascular hemodynamics and further increase the likelihood of cardiovascular events (40).

The findings of this meta-analysis support these observations: following sleep deprivation, cardiac vagal activity appears to be suppressed, as evidenced by a significant reduction in RMSSD. However, it is noteworthy that the literature on this topic remains somewhat inconsistent. During prolonged sleep deprivation, studies consistently show that sleep deprivation contributes to increased heart rate and enhanced sympathetic nervous system activity (1, 41, 42). However, in some studies, participants exhibited decreased heart rate and reduced cardiac sympathetic activity following sleep deprivation (43). These conflicting results may reflect individual differences in physiological adaptation and stress responses (44). For example, elevated psychological stress following prolonged sleep loss may increase cardiovascular reactivity, especially in older adults. This suggests that although sympathetic activity may decline in some cases, long-term stress adaptation may still result in a gradual reduction in heart rate over time.

In contrast, SDNN, as a comprehensive HRV parameter, reflects the overall cardio-autonomic modulation but is influenced by multiple factors, including experimental design, baseline physiological state, and individual compensatory mechanisms (45, 46). For example, some studies suggest that short-term sleep deprivation may not be sufficient to induce significant alterations in the overall fluctuations of sympathetic and parasympathetic activity, which may explain the non-significant changes in SDNN observed in this study (47).

From a mechanistic perspective, the HRV changes induced by sleep deprivation suggest that RMSSD is more sensitive in detecting acute physiological stress (44, 48). Unlike SDNN, the statistically significant reduction in RMSSD observed in this analysis highlights its superior sensitivity in capturing short-term autonomic nervous system responses, particularly in the context of acute sleep deprivation (49). However, despite reaching statistical significance, the effect size for RMSSD (SMD = −0.24) was relatively modest, indicating that the physiological impact of sleep deprivation on HRV may be limited or only significant under specific conditions (50, 77).

In summary, the results of this study support the use of RMSSD as a sensitive marker for detecting cardio-autonomic dysfunction induced by sleep deprivation, especially in acute physiological settings. Nevertheless, further research is needed to elucidate individual variability in HRV responses to sleep loss, as well as the cumulative effects of chronic sleep deprivation on cardiac autonomic regulation.

This study further analyzed the effects of sleep deprivation on frequency-domain HRV indicators. The results revealed that low-frequency power (LF) significantly increased following sleep deprivation (SMD = 0.39, 95% CI: 0.15–0.63, *p* = 0.002), and the LF/HF ratio also showed a significant rise (SMD = 1.47, 95% CI: 0.62–2.33, *p* = 0.0007). In contrast, high-frequency power (HF) demonstrated a downward trend, although the result did not reach statistical significance (SMD = −0.23, 95% CI: −0.46 to 0.01, *p* = 0.06).

Existing evidence suggests that an increase in LF is generally associated with enhanced sympathetic nervous activity, while changes in HF may be influenced by a variety of factors, including measurement conditions and individual variability (51–53). The significant elevation of the LF/HF ratio indicates a shift toward sympathovagal imbalance, reflecting relatively greater sympathetic dominance over vagal modulation. This pattern has been supported by prior studies, which showed that sympathetic activity increases markedly after 24 h of total sleep deprivation, leading to a notable rise in the LF/HF ratio (52, 54). The large effect size for LF/HF observed in this study further supports its sensitivity and utility in assessing dynamic autonomic alterations induced by sleep deprivation (51, 55). These results are consistent with the significant decrease in RMSSD observed in our analysis, together suggesting that sleep deprivation induces sympathetic predominance and remodeling of cardio-autonomic function.

However, it is important to note that non-neural factors—such as respiratory frequency, heart rate, and baroreflex sensitivity—can significantly influence both LF and HF values (21). Therefore, interpretations of the LF/HF ratio should be made with caution and should not be taken as a simple or direct measure of cardio-autonomic balance.

The meta-analysis for LF/HF revealed substantial heterogeneity across studies, prompting further subgroup analyses to identify potential sources. Subgrouping by sleep deprivation duration (<24 h vs. ≥24 h) showed high heterogeneity in both groups (*I*^2^ = 83 and 97%, respectively), suggesting that duration alone does not fully explain the observed inconsistency. Specifically, the <24 h subgroup yielded a significant effect size (SMD = 1.15, 95% CI: 0.32–1.97, *p* = 0.007), indicating that even short-term sleep deprivation can induce notable cardio-autonomic imbalance. The ≥24 h subgroup had an even larger effect size (SMD = 2.04), but the wide confidence interval (95% CI: −0.14 to 4.21, *p* = 0.07) and lack of statistical significance suggest instability in the pooled result, possibly due to one or two studies with disproportionate weight. This trend implies that longer durations of sleep deprivation do not necessarily amplify the sympathetic response in HRV, highlighting the need for further investigation into moderating variables.

Previous research has shown that following sleep deprivation, HF components (high-frequency power)—a marker of parasympathetic activity—tend to decrease significantly, while LF components (low-frequency power) may increase, indicating enhanced sympathetic nervous system activity (56). However, it is inappropriate to directly correlate the degree of sympathetic activation with the duration of sleep deprivation in a linear manner. Studies have demonstrated that prolonged sleep deprivation does not necessarily further amplify sympathetic responses, and may instead trigger other physiological adaptation mechanisms (57–59). In this study, although the ≥24-h subgroup had a larger effect size, its lack of statistical significance and high heterogeneity suggest that further investigation is needed to better understand how different durations of sleep deprivation affect cardio-autonomic function, both physiologically and psychologically.

In contrast, participant type appeared to be a more critical source of heterogeneity. The subgroup of university students showed no heterogeneity (*I*^2^ = 0%) and had the smallest effect size (SMD = 0.16, *p* = 0.57), whereas the subgroup of healthy general populations exhibited the highest heterogeneity (*I*^2^ = 91%) and a larger effect size (SMD = 1.77, *p* = 0.08). This notable difference indicates that sample homogeneity is key to ensuring the stability of meta-analytic results.

University student populations typically have narrow age ranges and similar lifestyle patterns, contribution to more consistent cardio-autonomic responses to acute sleep deprivation, reflected in smaller LF/HF elevations and lower data variability. In contrast, the “healthy population” subgroup encompasses a broader range of ages, occupations, and lifestyles, resulting in greater variability in sympathetic responses, which contributes to higher heterogeneity and larger effect sizes. However, due to incomplete data in the original studies, this meta-analysis could not formally assess other potential influencing factors such as age or lifestyle. Future studies are recommended to explore these aspects more thoroughly.

Special occupational groups such as resident physicians and miners also exhibited significantly elevated LF/HF ratios, indicating a strong cardio-autonomic activation response to sleep deprivation. This may be attributable to a baseline state of heightened sympathetic tension associated with long-term high-stress work environments, leading to an exaggerated or “overshooting” response when acute sleep deprivation is superimposed (60). Such activation may negatively impact cardiovascular function, including significant increases in heart rate and blood pressure (44). Furthermore, chronic sleep disruption inherent to these professions may have already reshaped their cardio-autonomic regulatory mechanisms, making their physiological responses to deprivation more pronounced.

This exaggerated response may also be partially explained by fluctuations in stress-related hormones, such as cortisol. Sleep deprivation is often accompanied by elevated levels of such hormones, which can disturb the balance of inflammatory markers (61). Chronic sleep loss may also alter the interplay between the central nervous system and the cardio-autonomic nervous system, making sympathetic activity even more sensitive to changes after deprivation (62). A key factor in this dynamic is the body’s acute stress response to occupational pressure and environmental demands. High-pressure work environments can result in a heightened endocrine and neural response to stressors. For example, one study found that within 24 h of sleep deprivation, endogenous levels of epinephrine and norepinephrine significantly increased, correlating with elevated heart rate and blood pressure—signs of a sustained state of sympathetic activation (63). This phenomenon is particularly evident in high-stress occupational groups who are chronically exposed to both stress and inadequate sleep. Such cumulative exposure may make their physiological responses to acute deprivation even more intense (64).

Although HF, as a specific marker of vagal (parasympathetic) activity, showed a declining trend, it did not reach statistical significance in this study. It is worth noting that HF is highly sensitive to respiratory rhythms, and thus may be indirectly influenced by changes in breathing patterns during sleep deprivation (65, 66). In addition, subtle differences in experimental design and implementation across studies may have contributed to the variability in HF findings, which warrants caution in interpretation (67, 68).

In summary, sleep deprivation was found to significantly affect frequency-domain HRV indicators—particularly LF and LF/HF—indicating enhanced sympathetic activity and suppressed parasympathetic tone. Participant-related heterogeneity appears to be the primary source of LF/HF variability, while the duration of sleep deprivation, though relevant, may not be the decisive factor. These findings highlight the need for stricter control of participant characteristics in future meta-analyses, as well as more nuanced interpretation that accounts for individual background and occupational exposure to avoid overgeneralization and result bias.

## 5 Limitations of this study

The number of included studies reporting HRV time-domain indicators such as SDNN and RMSSD was relatively limited, which may affect the reliability of the results. Some of the included studies had small sample sizes, which may lack sufficient statistical power to provide robust evidence. There was a wide age range among participants across studies, making it difficult to accurately assess the effects of sleep deprivation across different age groups. This study included only English and Chinese publications. Grey literature (e.g., military databases, dissertations, conference proceedings) was not included, due to both access limitations and concerns regarding peer-review quality and data consistency. This may have affected the completeness and reproducibility of the findings.

## 6 Conclusion

This meta-analysis provides evidence that sleep deprivation significantly affects autonomic nervous system regulation, as reflected in multiple HRV domains. (1) The time-domain indicator RMSSD showed a significant decrease following sleep deprivation, indicating suppression of cardiac vagal activity and increased sympathetic drive. (2) Frequency-domain indicators LF and LF/HF were significantly elevated, suggesting a disruption of sympatho-vagal balance and functional dysregulation of cardiac autonomic control.

However, divergent findings across studies—particularly regarding HF and other non-linear metrics—highlight the complexity of interpreting HRV under sleep-deprived conditions. These inconsistencies may stem from differences in computational approaches (e.g., time-domain vs. frequency-domain models), variations in physiological sensitivity among HRV parameters, and confounding factors such as sleep deprivation type (total vs. partial), measurement timing, and participant characteristics.

Therefore, while the current findings underscore the vulnerability of autonomic function to sleep deprivation, future research should consider integrating multi-dimensional HRV analysis and mechanistic physiological modeling to reconcile these conflicting results. This will enhance the translational value of HRV in evaluating cardiovascular risk under conditions of sleep loss.

## Data Availability

The original contributions presented in the study are included in the article/supplementary material, further inquiries can be directed to the corresponding authors.

## References

[1] Vierra J Boonla O Prasertsri P. Effects of sleep deprivation and 4-7-8 breathing control on heart rate variability, blood pressure, blood glucose, and endothelial function in healthy young adults. Physiol Rep. (2022) 10:e15389. doi: 10.14814/phy2.15389, PMID: 35822447 PMC9277512

[2] Wikanendra G Suhadi R Setiawan C Virginia D Hendra P Fenty F. Correlation among sleep duration, blood pressure, and blood glucose level of Morangan People, Sindumartani, Ngemplak, Sleman. J Pharm Sci Commun. (2020) 17:86–91. doi: 10.24071/jpsc.002404

[3] Egmond L Meth E Engström J Ilemosoglou M Keller J Vogel H . Effects of acute sleep loss on leptin, ghrelin, and adiponectin in adults with healthy weight and obesity: a laboratory study. Obesity. (2022) 31:635–41. doi: 10.1002/oby.23616 36404495

[4] Lim Y Hoe V Darus A Bhoo-Pathy N. Association between night-shift work, sleep quality and metabolic syndrome. Occup Environ Med. (2018) 75:716–23. doi: 10.1136/oemed-2018-105104, PMID: 30032104

[5] Shigiyama F Kumashiro N Tsuneoka Y Igarashi H Yoshikawa F Kakehi S . Mechanisms of sleep deprivation-induced hepatic steatosis and insulin resistance in mice. Am J Physiol Endocrinol Metab. (2018) 315:E848–58. doi: 10.1152/ajpendo.00072.2018, PMID: 29989853

[6] Periasamy S Chien S Hsu C Hsu D Liu M. Association between sleep deprivation and metformin treatment on pancreatic and liver function in mice. OBM Hepatol Gastroenterol. (2020) 4:1–18. doi: 10.21926/obm.hg.2002047

[7] Souza J Mônico-Neto M Tufik S Antunes H. Sleep debt and insulin resistance: what’s worse, sleep deprivation or sleep restriction? Sleep Sci. (2024) 17:e272–80. doi: 10.1055/s-0044-1782173, PMID: 39268336 PMC11390169

[8] Meyhöfer S Dembinski K Schultes B Born J Wilms B Lehnert H . Sleep deprivation prevents counterregulatory adaptation to recurrent hypoglycaemia. Diabetologia. (2022) 65:1212–21. doi: 10.1007/s00125-022-05702-9, PMID: 35445819 PMC9174142

[9] Younes M Gerardy B Pack A Kuna S Castro-Diehl C Redline S. Sleep architecture based on sleep depth and propensity: patterns in different demographics and sleep disorders and association with health outcomes. Sleep. (2022) 45:zsac059. doi: 10.1093/sleep/zsac059, PMID: 35272350 PMC9195236

[10] Taylor A Ed-Bansah D Tagoe T. Daytime sleepiness reflects depression, anxiety, and stress among students at the University of Ghana Medical School. Health Sci Investig J. (2023) 4:473–80. doi: 10.46829/hsijournal.2023.6.4.1.473-480

[11] Li W. Lack of sleep among college students can lead to negative emotions and affect memory. Lect Notes Educ Psychol Public Media. (2024) 45:16–22. doi: 10.54254/2753-7048/45/20230227

[12] Chang CH Wu HC Hsieh YR Lai WD Tung TH Huang JJ. Modulatory effect of n-3 polyunsaturated fatty acids on depressive-like behaviors in rats with chronic sleep deprivation: potential involvement of melatonin receptor pathway and brain lipidome. Food & function. (2023) 14:5977–5993. doi: 10.1039/d3fo01452e 37334912

[13] Li K Cardoso C Moctezuma-Ramirez A Elgalad A Perin E. Heart rate variability measurement through a smart wearable device: another breakthrough for personal health monitoring? Int J Environ Res Public Health. (2023) 20:7146. doi: 10.3390/ijerph20247146, PMID: 38131698 PMC10742885

[14] Andani A Arif W. The effect of lavender aromatherapy to improve the sleep quality of the elderly at Tresna Werdha Abiyoso Social Service Center, Sleman. J World Future Med Health Nurs. (2023) 1:163–70. doi: 10.55849/health.v1i3.518

[15] Xue Y Tang J Zhang M He Y Fu J Ding F. Durative sleep fragmentation with or without hypertension suppress rapid eye movement sleep and generate cerebrovascular dysfunction. Neurobiol Dis. (2023) 184:106222. doi: 10.1016/j.nbd.2023.106222, PMID: 37419254

[16] Moric-Janiszewska E Smolik S Szydłowski L Kapral M. Associations between selected *ADRB*1 and *CYP2D6* gene polymorphisms in children with ventricular and supraventricular arrhythmias. Medicina. (2023) 59:2057. doi: 10.3390/medicina59122057, PMID: 38138160 PMC10744405

[17] Correia ATL Lipinska G Rauch HGL Forshaw PE Roden LC Rae DE. Associations between sleep-related heart rate variability and both sleep and symptoms of depression and anxiety: a systematic review. Sleep Med. (2023) 101:106–17. doi: 10.1016/j.sleep.2022.10.018, PMID: 36370515

[18] Tiwari R Kumar R Malik S Raj T Kumar P. Analysis of heart rate variability and implication of different factors on heart rate variability. Curr Cardiol Rev. (2021) 17:e160721189770. doi: 10.2174/1573403X16999201231203854, PMID: 33390146 PMC8950456

[19] Godijk N Vos A Jongen V Moraba R Tempelman H Grobbee D . Heart rate variability, HIV and the risk of cardiovascular diseases in rural South Africa. Glob Heart. (2020) 15:17. doi: 10.5334/gh.532, PMID: 32489790 PMC7218774

[20] Hadad R Larsen B Weber P Stavnem D Kristiansen O Nielsen O . Night-time heart rate variability identifies high-risk people among people with uncomplicated type 2 diabetes mellitus. Diabet Med. (2021) 38:e14559. doi: 10.1111/dme.14559, PMID: 33714218

[21] Billman GE. The LF/HF ratio does not accurately measure cardiac sympatho-vagal balance. Front Physiol. (2013) 4:26. doi: 10.3389/fphys.2013.00026, PMID: 23431279 PMC3576706

[22] Zhong X Hilton HJ Gates GJ Jelic S Stern Y Bartels MN . Increased sympathetic and decreased parasympathetic cardiovascular modulation in normal humans with acute sleep deprivation. J Appl Physiol. (2005) 98:2024–32. doi: 10.1152/japplphysiol.00620.2004, PMID: 15718408

[23] Tarvainen MP Niskanen JP Lipponen JA Ranta-Aho PO Karjalainen PA. Kubios HRV—heart rate variability analysis software. Comput Methods Prog Biomed. (2014) 113:210–20. doi: 10.1016/j.cmpb.2013.07.024, PMID: 24054542

[24] Kumar SM Vaishali K Maiya GA Shivashankar KN Shashikiran U. Analysis of time-domain indices, frequency domain measures of heart rate variability derived from ECG waveform and pulse-wave-related HRV among overweight individuals: an observational study. F1000Res. (2023) 12:1229. doi: 10.12688/f1000research.139283.1, PMID: 37799491 PMC10548108

[25] Shaffer F Ginsberg JP. An overview of heart rate variability metrics and norms. Front Public Health. (2017) 5:258. doi: 10.3389/fpubh.2017.00258, PMID: 29034226 PMC5624990

[26] Heathers JA. Everything hertz: methodological issues in short-term frequency-domain HRV. Front Physiol. (2014) 5:177. doi: 10.3389/fphys.2014.00177, PMID: 24847279 PMC4019878

[27] Higgins JP Altman DG Gøtzsche PC Jüni P Moher D Oxman AD . The Cochrane Collaboration’s tool for assessing risk of bias in randomised trials. BMJ. (2011) 343:d5928. doi: 10.1136/bmj.d5928, PMID: 22008217 PMC3196245

[28] Cheung MW Vijayakumar R. A guide to conducting a meta-analysis. Neuropsychol Rev. (2016) 26:121–8. doi: 10.1007/s11065-016-9319-z, PMID: 27209412

[29] Page MJ McKenzie JE Bossuyt PM Boutron I Hoffmann TC Mulrow CD . The PRISMA 2020 statement: an updated guideline for reporting systematic reviews. BMJ. (2021) 372:n71. doi: 10.1136/bmj.n71, PMID: 33782057 PMC8005924

[30] Garbarino S Lanteri P Bragazzi N Magnavita N Scoditti E. Role of sleep deprivation in immune-related disease risk and outcomes. Commun Biol. (2021) 4:1304. doi: 10.1038/s42003-021-02825-4, PMID: 34795404 PMC8602722

[31] Nollet M Wisden W Franks N. Sleep deprivation and stress: a reciprocal relationship. Interface Focus. (2020) 10:20190092. doi: 10.1098/rsfs.2019.0092, PMID: 32382403 PMC7202382

[32] Lyons L Chatterjee S Vanrobaeys Y Gaine M Abel T. Translational changes induced by acute sleep deprivation uncovered by Trap-Seq. Mol Brain. (2020) 13:165. doi: 10.1186/s13041-020-00702-5, PMID: 33272296 PMC7713217

[33] Lyons L Vanrobaeys Y Abel T. Sleep and memory: the impact of sleep deprivation on transcription, translational control, and protein synthesis in the brain. J Neurochem. (2023) 166:24–46. doi: 10.1111/jnc.15787, PMID: 36802068 PMC10919414

[34] Gaine M Bahl E Chatterjee S Michaelson J Abel T Lyons L. Altered hippocampal transcriptome dynamics following sleep deprivation. Mol Brain. (2021) 14:125. doi: 10.1186/s13041-021-00835-1, PMID: 34384474 PMC8361790

[35] Su Q Jiang D Zhong Z Zhou K Gong W. Chinese medicine Jiangzhuo mixture regulates glucose and lipid metabolism in obese rats through TLR4/IκBα/NF-κB signaling pathway. J Zhejiang Univ Med Sci. (2023) 52:627–35. doi: 10.3724/zdxbyxb-2023-0164, PMID: 37899401 PMC10630061

[36] Tobaldini E Cogliati C Fiorelli EM Nunziata V Wu MA Prado M . One night on-call: sleep deprivation affects cardiac autonomic control and inflammation in physicians. Eur J Intern Med. (2013) 24:664–70. doi: 10.1016/j.ejim.2013.03.011, PMID: 23601527

[37] Chung MH Kuo TB Hsu N Chu H Chou KR Yang CC. Recovery after three-shift work: relation to sleep-related cardiac neuronal regulation in nurses. Ind Health. (2012) 50:24–30. doi: 10.2486/indhealth.ms1305, PMID: 22146144

[38] Mullington JM Haack M Toth M Serrador JM Meier-Ewert HK. Cardiovascular, inflammatory, and metabolic consequences of sleep deprivation. Prog Cardiovasc Dis. (2009) 51:294–302. doi: 10.1016/j.pcad.2008.10.003, PMID: 19110131 PMC3403737

[39] Wang ML Lin PL Huang CH Huang HH. Decreased parasympathetic activity of heart rate variability during anticipation of night duty in anesthesiology residents. Anesth Analg. (2018) 126:1013–8. doi: 10.1213/ANE.0000000000002439, PMID: 29200073

[40] Dardi P Costa D Assunção H Rossoni L. Venous endothelial function in cardiovascular disease. Biosci Rep. (2022) 42:BSR20220285. doi: 10.1042/bsr20220285 36281946 PMC9685499

[41] Tobaldini E Covassin N Calvin A Singh P Bukartyk J Wang S . Cardiac autonomic control and complexity during sleep are preserved after chronic sleep restriction in healthy subjects. Physiol Rep. (2017) 5:e13197. doi: 10.14814/phy2.13197, PMID: 28408635 PMC5392506

[42] Wehrens SM Hampton SM Skene DJ. Heart rate variability and endothelial function after sleep deprivation and recovery sleep among male shift and non-shift workers. Scand J Work Environ Health. (2012) 38:171–81. doi: 10.5271/sjweh.3197, PMID: 21953310

[43] Huang XT. The Effect of acute partial sleep deprivation on cardiovascular autonomic nervous activity in medical staff In: Dissertation. Fuzhou: Fujian Medical University (2017)

[44] Franzen P Gianaros P Marsland A Hall M Siegle G Dahl R . Cardiovascular reactivity to acute psychological stress following sleep deprivation. Psychosom Med. (2011) 73:679–82. doi: 10.1097/psy.0b013e31822ff440, PMID: 21949422 PMC3614084

[45] Gong M Min S Sun Y Jin L Li S. Effects of acute sleep deprivation on sporting performance in athletes: a comprehensive systematic review and meta-analysis. Nat Sci Sleep. (2024) 16:935–48. doi: 10.2147/nss.s467531 39006249 PMC11246080

[46] Havekes R Park A Tudor J Luczak V Hansen R Ferri S . Sleep deprivation causes memory deficits by negatively impacting neuronal connectivity in hippocampal area CA1. eLife. (2016) 5:e13424. doi: 10.7554/elife.13424, PMID: 27549340 PMC4996653

[47] Basner M Rao H Goel N Dinges D. Sleep deprivation and neurobehavioral dynamics. Curr Opin Neurobiol. (2013) 23:854–63. doi: 10.1016/j.conb.2013.02.008, PMID: 23523374 PMC3700596

[48] Bourdillon N Jeanneret F Nilchian M Albertoni P Ha P Millet GP. Sleep deprivation deteriorates heart rate variability and photoplethysmography. Front Neurosci. (2021) 15:642548. doi: 10.3389/fnins.2021.642548, PMID: 33897355 PMC8060636

[49] Shao Y Xu L Peng Z An X Gong J Han M. Non-linear effects of acute sleep deprivation on spatial working memory: cognitive depletion and neural compensation. Brain Sci. (2024) 15:18. doi: 10.3390/brainsci15010018, PMID: 39851387 PMC11763834

[50] Umemura G Pinho J Duysens J Krebs H Forner-Cordero A. Sleep deprivation affects gait control. Sci Rep. (2021) 11:21104. doi: 10.1038/s41598-021-00705-9, PMID: 34702960 PMC8548553

[51] Ghazali A Warif N Yazit N Juliana N Ishak I Ibrahim F . Quran memorisation and heart rate variability: how do they correlate? World J Clin Cases. (2024) 12:6275–84. doi: 10.12998/wjcc.v12.i29.6275 39417055 PMC11372528

[52] Lombardi F Stein P. Origin of heart rate variability and turbulence: an appraisal of autonomic modulation of cardiovascular function. Front Physiol. (2011) 2:95. doi: 10.3389/fphys.2011.00095, PMID: 22163222 PMC3233900

[53] Vanderlei L Pastre C Hoshi R Carvalho T Godoy M. Noções básicas de variabilidade da frequência cardíaca e sua aplicabilidade clínica. Braz J Cardiovasc Surg. (2009) 24:205–17. doi: 10.1590/s0102-76382009000200018

[54] Huang W Hwang B Lai C Li J Kuo T Yang C. Is heart rate variability related to season of birth? Clin Cardiol. (2015) 38:407–12. doi: 10.1002/clc.22410, PMID: 26212374 PMC6711004

[55] Juszczak K Mazur M Wyczokowski M Filipek M Thor P. Autonomic nervous system activity in patients with lower urinary tract symptoms secondary to benign prostatic hyperplasia estimated by heart rate variability. Open Urol Nephrol J. (2008) 1:44–9. doi: 10.2174/1874303x00801010044

[56] Trinder J Kleiman J Carrington M Smith S Breen S Tan N . Autonomic activity during human sleep as a function of time and sleep stage. J Sleep Res. (2001) 10:253–64. doi: 10.1046/j.1365-2869.2001.00263.x, PMID: 11903855

[57] Ako M Kawara T Uchida S Miyazaki S Nishihara K Mukai J . Correlation between electroencephalography and heart rate variability during sleep. Psychiatry Clin Neurosci. (2003) 57:59–65. doi: 10.1046/j.1440-1819.2003.01080.x, PMID: 12519456

[58] Kesek M Franklin K Sahlin C Lindberg E. Heart rate variability during sleep and sleep apnoea in a population based study of 387 women. Clin Physiol Funct Imaging. (2009) 29:309–15. doi: 10.1111/j.1475-097x.2009.00873.x, PMID: 19453563

[59] Okamoto-Mizuno K Yamashiro Y Tanaka H Komada Y Mizuno K Tamaki M . Heart rate variability and body temperature during the sleep onset period. Sleep Biol Rhythms. (2008) 6:42–9. doi: 10.1111/j.1479-8425.2008.00335.x

[60] Carter J Durocher J Larson R DellaValla J Yang H. Sympathetic neural responses to 24-hour sleep deprivation in humans: sex differences. Am J Phys Heart Circ Phys. (2012) 302:H1991–7. doi: 10.1152/ajpheart.01132.2011, PMID: 22408018 PMC3362107

[61] Wright K Drake A Frey D Fleshner M DeSouza C Gronfier C . Influence of sleep deprivation and circadian misalignment on cortisol, inflammatory markers, and cytokine balance. Brain Behav Immun. (2015) 47:24–34. doi: 10.1016/j.bbi.2015.01.004, PMID: 25640603 PMC5401766

[62] Perry J Bergamaschi C Campos R Silva A Tufik S. Interconnectivity of sympathetic and sleep networks is mediated through reduction of gamma aminobutyric acidergic inhibition in the paraventricular nucleus. J Sleep Res. (2013) 23:168–75. doi: 10.1111/jsr.12110, PMID: 24283672

[63] Xu Y Qu B Liu F Gong Z Zhang Y Xu D. Sleep deprivation and heart rate variability in healthy volunteers: effects of rem and SWS sleep deprivation. Comput Math Methods Med. (2023) 2023:7121295. doi: 10.1155/2023/7121295, PMID: 37469834 PMC10353901

[64] Li J Zhang H Deng B Wang X Peng L Xu S . Dexmedetomidine improves anxiety-like behaviors in sleep-deprived mice by inhibiting the p38/msk1/nfκb pathway and reducing inflammation and oxidative stress. Brain Sci. (2023) 13:1058. doi: 10.3390/brainsci13071058 37508990 PMC10377202

[65] Medić G Wille M Hemels M. Short- and long-term health consequences of sleep disruption. Nat Sci Sleep. (2017) 9:151–61. doi: 10.2147/nss.s134864, PMID: 28579842 PMC5449130

[66] Tak Y Lee J Kim Y Lee S Cho B. 25-hydroxyvitamin d and its relationship with autonomic dysfunction using time- and frequency-domain parameters of heart rate variability in Korean populations: a cross-sectional study. Nutrients. (2014) 6:4373–88. doi: 10.3390/nu6104373, PMID: 25325256 PMC4210923

[67] Ekici B. The effects of the duration of mobile phone use on heart rate variability parameters in healthy subjects. Anatol J Cardiol. (2016) 16:833–8. doi: 10.14744/anatoljcardiol.2016.6717, PMID: 27109242 PMC5324882

[68] Ji H Woo H Park Y Hwang D Lee J Lee C . Characteristics of heart rate variability in women with polycystic ovary syndrome. Medicine. (2018) 97:e12510. doi: 10.1097/md.0000000000012510, PMID: 30235765 PMC6160158

[69] Liu Q Zhou R Liu L Zhao X. Effects of 72 hours total sleep deprivation on male astronauts’ executive functions and emotion. Compr Psychiatry. (2015) 61:28–35. doi: 10.1016/j.comppsych.2015.05.015, PMID: 26112064

[70] Morales J Yáñez A Fernández-González L Montesinos-Magraner L Marco-Ahulló A Solana-Tramunt M . Stress and autonomic response to sleep deprivation in medical residents: a comparative cross-sectional study. PLoS One. (2019) 14:e0214858. doi: 10.1371/journal.pone.0214858, PMID: 30947295 PMC6448892

[71] van Leeuwen WMA Sallinen M Virkkala J Lindholm H Hirvonen A Hublin C . Physiological and autonomic stress responses after prolonged sleep restriction and subsequent recovery sleep in healthy young men. Sleep Biol Rhythms. (2018) 16:45–54. doi: 10.1007/s41105-017-0122-x, PMID: 29367834 PMC5754428

[72] Kunikullaya KU Kirthi SK Venkatesh D Goturu J. Heart rate variability changes in business process outsourcing employees working in shifts. Indian Pacing Electrophysiol J. (2010) 10:439–46. PMID: 21151382 PMC2974331

[73] Ma L Zheng LP Wu YY Zhang D Ma J Wang HY . Observation on the effects of sleep deprivation on electrocardiographic activity in naval personnel during maiden voyage. People’s Mil Surg. (2014) 57:1296–9.

[74] Guan CC Shen C Cheng S Zhang TH Ma J Hu WD. A study on the recovery of task performance by physiological electrical stimulation in a sleep deprivation fatigue model. Occup Health. (2020) 36:2676–81. doi: 10.13329/j.cnki.zyyjk.2020.0708

[75] Wu JF Wu Q Sun SQ. Heart rate variability in simulated driving fatigue conditions. Zhonghua Lao Dong Wei Sheng Zhi Ye Bing Za Zhi. (2010) 28:686–8. doi: 10.3760/cma.j.issn.1001-9391.2010.09.015 21126486

[76] Byun S Kim A Jang E Kim S Choi K Yu H . Entropy analysis of heart rate variability and its application to recognize major depressive disorder: a pilot study. Technol Health Care. (2019) 27:407–24. doi: 10.3233/thc-199037, PMID: 31045557 PMC6597986

[77] Jung I Lee D Lee M Kwon H Rhee E Park C . Autonomic imbalance increases the risk for non-alcoholic fatty liver disease. Front Endocrinol. (2021) 12:752944. doi: 10.3389/fendo.2021.752944, PMID: 34819920 PMC8606663

[78] Kazé A Yuyun M Ahima R Sachdeva M Echouffo-Tcheugui J. Association of heart rate variability with progression of retinopathy among adults with type 2 diabetes. Diabet Med. (2022) 39:e14857. doi: 10.1111/dme.14857, PMID: 35467041 PMC9324816

[79] Li NW Fang XK. A study on the effects of sleep deprivation on risk perception in miners: based on a physiological experiment. J Saf Environ. (2024) 24:1036–43. doi: 10.13637/j.issn.1009-6094.2023.0444

[80] Li P Feng J Guo J Xue J Li Y Wen S . Reduced expression of the PER2 protein contributes to β 1-AA-induced cardiac autophagy rhythm disorders. Acta Biochim Biophys Sin. (2025) 56:1–10. doi: 10.3724/abbs.2025023 PMC12536457 40211826

[81] Luo F Han S Xia M Chen Z Liu P Lin J. Study on the mechanism of hydrolyzed seawater pearl tablet in treating chronic sleep deprivation mice model. Endocr Metab Immune Disord Drug Targets. (2023) 23:927–36. doi: 10.2174/1871530323666230206160722, PMID: 36748223 PMC10278239

[82] Lins-Filho OL Andrade-Lima A Torres AD Oliveira LM Luiz do-Prado W Ritti-Dias R . Association between sleep quality and cardiac autonomic modulation in adolescents: a cross sectional study. Sleep Sci. (2023) 16:e462–7. doi: 10.1055/s-0043-1776750, PMID: 38197026 PMC10773521

[83] Liou J Wang P Wu Y Lee S Chang S Liou M. ECG approximate entropy in the elderly during cycling exercise. Sensors. (2022) 22:5255. doi: 10.3390/s22145255, PMID: 35890935 PMC9324578

[84] Seidel F Scheibenbogen C Heidecke H Opgen-Rhein B Pickardt T Klingel K . Compensatory upregulation of anti-beta-adrenergic receptor antibody levels might prevent heart failure presentation in pediatric myocarditis. Front Pediatr. (2022) 10:881208. doi: 10.3389/fped.2022.881208, PMID: 35573966 PMC9096696

[85] Snelder S Aga Y Laat L Biter L Cabezas M Pouw N . Normalization of cardiac function after bariatric surgery is related to autonomic function and vitamin D. Obes Surg. (2022) 33:47–56. doi: 10.1007/s11695-022-06336-x 36334252 PMC9834145

[86] Chen PH Chung CC Liu SH Kao YH Chen YJ. Lithium Treatment Improves Cardiac Dysfunction in Rats Deprived of Rapid Eye Movement Sleep. International journal of molecular sciences. (2022) 23:11226–56. doi: 10.3390/ijms231911226 36232526 PMC9570242

